# Post-vaccination SARS-CoV-2 neutralizing antibodies in pregnant women receiving biologics for inflammatory bowel disease

**DOI:** 10.1371/journal.pone.0321242

**Published:** 2025-12-03

**Authors:** Donna E. Leet, Jing Jin, Charles S. Craik, Michael G. Kattah, Millie D. Long, Uma Mahadevan

**Affiliations:** 1 Department of Medicine, University of California, San Francisco, California, United States of America; 2 Vitalant Research Institute, San Francisco, California, United States of America; 3 Department of Pharmaceutical Chemistry, University of California, San Francisco, California, United States of America; 4 Department of Medicine, University of North Carolina, Chapel Hill, North Carolina, United States of America; Centers for Disease Control and Prevention, UNITED STATES OF AMERICA

## Abstract

Inflammatory bowel disease (IBD) treatments and pregnancy can independently modulate immune responses, but the combined impacts on SARS-CoV-2 vaccine-induced immunity are poorly understood. This study explores the efficacy of SARS-CoV-2 vaccination and placental antibody transfer among pregnant women with IBD on biologic therapies. This observational study included pregnant women with and without IBD from the PIANO and PREVENT-COVID studies and their neonates. We assessed anti-SARS-CoV-2 neutralizing antibody titers (NT50) in maternal and cord blood post-vaccination using a pseudotype neutralization assay and calculated placental transfer ratios. A total of 32 pregnant women participated, and 27 were exposed to a biologic medication during pregnancy. Neutralizing antibody titers were similar between biologic-treated and non-treated groups, and biologic-exposed women demonstrated robust placental transfer of neutralizing antibodies. Corticosteroid use during pregnancy was significantly associated with reduced placental transfer efficiency, although this effect was not significant in a sensitivity analysis excluding patients treated with immunomodulators. Vaccination timing and SARS-CoV-2 infection impacted maternal and cord antibody levels, with higher titers observed in those vaccinated before pregnancy or infected during pregnancy. Overall, our findings suggest that upon vaccination, pregnant women with IBD on biologic therapies mount effective SARS-CoV-2 neutralizing antibody responses similar to their non-biologic-exposed counterparts, with efficient placental transfer. These findings support the efficacy of SARS-CoV-2 vaccination in this population, though further research with larger cohorts is needed for validation and to explore the long-term protective effects of transferred antibodies in neonates. Furthermore, corticosteroid use, immunomodulator use, and vaccination timing may influence antibody dynamics, underscoring the need for tailored clinical management in this vulnerable population.

## Introduction

Effective SARS-CoV-2 vaccination can significantly reduce the risk of COVID infection and mortality in patients with inflammatory bowel disease (IBD) while also improving neonatal outcomes [1]. However, pregnancy introduces unique immunological adaptations, and when combined with immunosuppressive therapies commonly used to manage IBD, these factors may cumulatively influence the immune response to vaccination. These therapies, particularly biologics, may alter vaccine-induced immunity and potentially affect the efficiency of antibody transfer from mother to fetus. Understanding these combined effects is essential to guide clinical recommendations, reassure patients, and optimize the timing and approach to vaccination in this vulnerable population. However, limited data exist on the efficacy of SARS-CoV-2 vaccination in pregnant women with IBD receiving immunosuppressive therapy, particularly regarding the placental transfer of antibodies.

While pregnant and non-pregnant women have shown similar antibody responses to SARS-CoV-2 vaccination [2], several studies have illustrated reduced vaccine responses in patients with IBD taking anti-tumor necrosis factor (TNF) agents or Jak kinase (JAK) inhibitors [3,4]. Moreover, placental transfer of COVID antibodies in healthy pregnant women has been established to varying degrees [5,6], but whether immunosuppressive therapies impact this process is unknown. Finally, most prior studies have quantified SARS-CoV-2 immunoglobulin levels rather than the examining the quality of antibodies denoted by the neutralizing antibody titer, which is of significant biological relevance in patient outcomes [7]. To address these knowledge gaps, we studied a cohort of pregnant women with and without IBD and evaluated the impact of biologic use on (1) anti-SARS-CoV-2 neutralizing antibody titers in post-vaccination maternal and cord blood and (2) placental transfer of anti-SARS-CoV-2 neutralizing antibodies.

## Methods

### Study design and patient recruitment

The study was conducted according to Declaration of Helsinki principles and was approved by the Institutional Review Board of the University of California, San Francisco (#10-00831). Written informed consent was received from participants prior to inclusion in the study. The Pregnancy in Inflammatory Bowel Disease and Neonatal Outcomes (PIANO) study is an ongoing prospective observational study that has enrolled over 2018 pregnant women with and without IBD at more than 30 US centers beginning in January 2007. Partnership to Report Effectiveness of Vaccination in populations Excluded from iNitial Trials of COVID (PREVENT-COVID) is a prospective, observational, cohort study of patients with IBD in the United States who have received any COVID-19 vaccine that has enrolled 3505 participants with IBD from across the US. Between February 8^th^ 2021 and December 31^st^ 2022, pregnant women in the PIANO study, the PREVENT-COVID study, or who were seen within the UCSF Gastroenterology clinic and who had received at least one SARS-CoV-2 vaccination were deemed eligible and recruited. Using patient questionnaires and medical records, we collected (1) maternal demographic variables including age, body mass index, parity, self-reported race/ethnicity, gestational age at delivery and smoking, (2) COVID-related variables including type of vaccination, dates of vaccination(s) before and during pregnancy, incidence and timing of COVID infection during pregnancy, and (3) IBD-related variables including diagnosis, disease activity, and IBD medications (S1 File). Information on vaccination-related adverse events was not collected. Serum was collected from women, umbilical cords, and in some cases, newborns, at the time of delivery. The sample size in the comparison (non-biologic) arm was limited due to challenges in recruiting patients and their neonates after 2022.

### Laboratory analysis

#### Production of pseudoviruses for the SARS-CoV-2 neutralization assay.

Serum samples were assayed for the presence of anti-SARS-CoV-2 neutralizing antibodies at UCSF or at Vitalant Research Institute (San Francisco, California) using a lentivirus-based pseudotype neutralization assay [8]. Briefly, VSVΔG-luciferase-based pseudotype reporter viruses, in which the VSV glycoprotein (VSV-G) gene is replaced with firefly luciferase gene, were generated by transducing cells expressing viral glycoproteins with VSV-G/ VSVΔG-Luc pseudoviruses. BHK-21 cells were transfected with pCAGGS SARS-CoV-2 SΔ21 that expresses codon-optimized SARS-CoV-2 spike with the C-terminal 21 amino acids deleted to remove the ER-retrieval signal. At ~16 hours post-transfection, cells were treated with 3.75 mM valproic acid for 4 hours, then were transduced with VSV-G/ VSVΔG-Luc at a multiplicity of infection of 0.3. Six hours later, cells were washed and incubated with fresh medium containing anti-VSV-G to neutralize any residual VSV-G/ VSVΔG-Luc. At ~24 hours post-transduction, the supernatants containing SARS-CoV-2 Spike pseudotyped VSV-G/ VSVΔG-Luc reporter viruses (SARS-CoV-2 S/ VSVΔG-Luc) were harvested and filtered through a 0.45 µm filter to remove cell debris.

#### Neutralization of SARS-CoV-2 spike-mediated infection.

HEK293T cells stably expressing human ACE2 and TMPRSS2 (293T-ACE2-TMPRSS2 cells) were used as the target cells in the neutralization assay. 293T-ACE2-TMPRSS2 cells were seeded in 96-well-plates at a density of 3 x 10^4^ cells/well overnight. Plasma or serum samples were 4-fold serially diluted from 1:20 to 1:5,120. SARS-CoV-2 S/ VSVΔG-Luc reporter viruses were mixed with serially diluted plasm or serum at 1:1 ratio and incubated at 37 °C for 1 hour. Subsequently, 293T-ACE2-TMPRSS2 cells were infected with 100 µl reporter virus/plasma or serum mix per well. At 16–24 hours post-infection, cells were lysed for luciferase activity measurement with the luciferase assay system (Promega) following manufacturer instructions. Percentage of infectivity was calculated as a percentage of no plasma/serum control. Nonlinear regression curves, 50% neutralization titers (NT50) and 80% neutralization titers (NT80) were calculated in GraphPad Prism (v10.2.3).

### Statistical analysis

Data analysis was conducted using GraphPad Prism (v10.2.3). Placental antibody transfer ratios were calculated as the cord blood NT50 divided by maternal NT50. Vaccine latency was defined as the time between most recent vaccination date and sample collection date (i.e., delivery date). We used descriptive statistics to characterize biologic-exposed and non-exposed populations, with continuous data reported as median and interquartile range (IQR), and descriptive data as numbers and percentages, unless otherwise stated. For comparison of the demographics data between biologic-exposed and non-exposed patients, we used Mann-Whitney U tests for continuous data and Fisher’s exact tests for descriptive data. For the univariate analysis, the Shapiro-Wilk test was used to determine data distribution normality. The data sets were shown to be non-normally distributed. Therefore, the Kruskal-Wallis test was performed to determine significant differences between three or more groups and the Mann-Whitney U test was performed to compare two groups. Spearman’s rank correlation coefficient was calculated to determine the relationship between two sets of non-parametric data. For multiple comparisons, a two-stage step-up method of Benjamini, Krieger, and Yekutieli was applied, using a false-discovery-rate (FDR) of 0.10. To evaluate whether the sample size was sufficient to detect differences in neutralizing antibody titers (NT50) and transfer ratios between biologic-exposed and non-exposed pregnant individuals, we conducted a post hoc power analysis using the R package pwr. We first calculated the effect size (Cohen’s *d*) based on values from biologic-exposed (n = 27) and non-exposed (n = 5) patients. Power was then estimated using a two-sample *t*-test (function pwr.t.test) assuming a two-sided test with α = 0.05. Two-tailed p-values less than 0.05 were considered significant.

## Results

### Demographics

A total of 96 women were confirmed eligible and recruited for the study, of which 32 patients enrolled, and 29 had IBD. A total of 27 women were on a biologic during pregnancy. The majority of biologic-treated women were on an anti-TNF agent (70.4%). Of the two women with IBD not treated with a biologic, one was treated with mesalamine, and one received azathioprine during pregnancy. Three healthy women without IBD were also included in the no biologic cohort. Cord blood was not collected from two patients. None of the participants had significant cardiopulmonary or metabolic comorbidities. For the IBD group, the mothers’ median age at delivery was 34.4 years, and median gestational age at delivery was 39.6 weeks. As expected, the breakdown of disease states between the non-biologic and biologic-exposed cohorts were statistically disparate, but there were no significant differences in pregnancy-related, SARS-CoV-2-related, and other IBD-related demographic characteristics between biologic-exposed and non-exposed cohorts (Table 1).

**Table 1:** Cohort demographics.

	Biologic-Exposed	Non-Biologic- Exposed	
	n = 27	n = 5	p-value
Maternal age at delivery, years	34.4 (31.3–36.6)	36.2 (34.9–39.0)	0.11
BMI prior to pregnancy	22.0 (20.7–24.3)	22.3 (20.8–24.6)	0.95
Smoking history			
Never	22 (81.5)	5 (100)	
Past/present	5 (18.5)	0 (0)	0.56
Disease state			
Healthy	0 (0)	3 (60)	
Crohn’s Disease	17 (63.0)	0 (0)	
Ulcerative Colitis	10 (37.0)	2 (40)	<0.001
Gestational age at delivery, weeks	39.6 (39–40)	39.0 (35.2–40.4)	0.28
Parity	1 (1–2)	2 (1–2)	0.66
Type of delivery			
Vaginal	22 (81.5)	3 (60)	
Caesarean	5 (18.5)	2 (40)	0.3
Type of biologic received during pregnancy			
Anti-TNF	19 (70.4)		
Anti-IL12/23	8 (29.6)	n/a	n/a
Steroid exposure during pregnancy	4 (14.8)	0 (0)	>0.99
Immunomodulator exposure during pregnancy	4 (14.8)	1 (20)	>0.99
Vaccinated during pregnancy	18 (66.7)	4 (80)	>0.99
COVID infection during pregnancy	7 (25.9)	1 (20)	>0.99
Time from last COVID vaccine or infection to antibody testing, days	141 (54–228)	98 (51.5–153.5)	0.45
Time from last COVID vaccine to antibody testing, days	156 (56.5–370)	98 (51.5–255.5)	0.76
Vaccine type/ Bivalent reception			
BNT162b2 mRNA	14 (51.9)	5 (100)	
mRNA-1273	13 (48.1)	0 (0)	0.06
Received Bivalent	4 (14.8)	0 (0)	>0.99
Active disease	1 (3.7)	0 (0)	>0.99

Table 1 data are reported as median (IQR) or number (%). Mann-Whitney U tests were used for continuous data and Fisher’s exact tests for descriptive data.

### Maternal and cord blood neutralizing antibody responses

**Fig 1:**
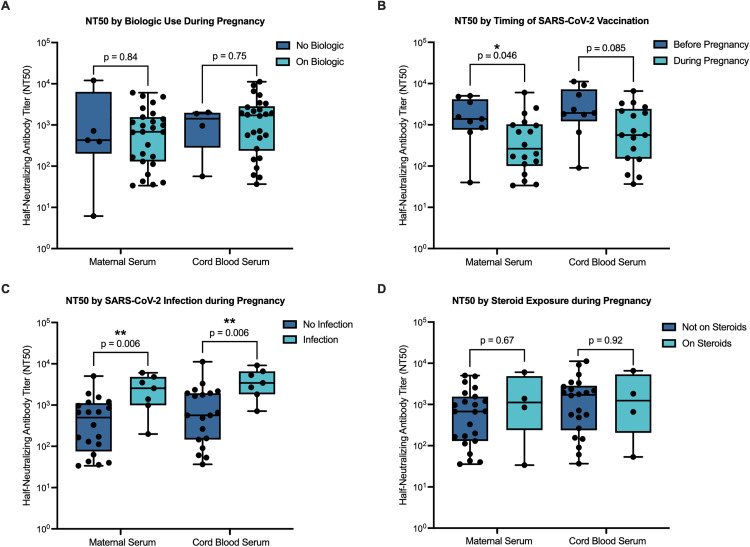
Impact of biologic exposure, timing of vaccination, SARS-CoV-2 infection, and corticosteroid use on maternal and cord blood anti-SARS-CoV-2 neutralizing antibody titers. (A) Maternal and cord blood neutralizing antibody titers (NT50) against SARS-CoV-2 by biologic exposure during pregnancy. (B) NT50 by timing of SARS-CoV-2 vaccination completion in relation to pregnancy in biologic-exposed patients. (C) NT50 by incidence of SARS-CoV-2 infection during pregnancy in biologic-exposed patients. (D) NT50 by steroid exposure during pregnancy in biologic-exposed patients. Cord blood was not collected for one biologic-exposed patient and one non-biologic-exposed patient with IBD. Mann-Whitney U tests were performed.

There was no difference in maternal nor cord blood anti-SARS-CoV-2 neutralizing antibody titers (NT50) at delivery between biologic-exposed and non-exposed women (Fig 1A). Maternal and cord blood NT50 also did not vary by biologic type (anti-TNF exposure versus anti-IL12/23 exposure, not shown). Univariate analysis of all patients as well as the biologic-exposed subgroup revealed higher maternal NT50, and a trend toward higher cord blood NT50, in women who completed SARS-CoV-2 vaccination prior to conception as opposed to during pregnancy, although the higher maternal NT50 was not significant after adjustment for multiple comparisons (Fig 1B and not shown). Analogously, in biologic-exposed women, there was a positive correlation between time elapsed since final vaccine dose and maternal and cord blood neutralizing antibody titers, with higher NT50 associated with longer latency periods (Table 2).

**Table 2:** Neutralizing antibody titers and transfer ratio by vaccine latency and gestational age.

	Maternal NT50		Cord Blood NT50		Transfer Ratio	
	Spearman r	p-value	Spearman r	p-value	Spearman r	p-value
**Vaccine Latency** **(All Patients)**	0.34	0.06	0.42	**0.02**	0.16	0.4
**Vaccine Latency** **(Biologic-exposed patients)**	0.42	**0.03**	0.40	**0.04**	0.10	0.63
**Vaccine Latency** (All Patients, vaccinated during pregnancy)*	0.12	0.59	0.30	0.21	0.50	**0.03**
Gestational Age at Last Vaccination (All patients*)	−0.06	0.80	−0.20	0.41	−0.49	**0.03**
Gestational Age at Last Vaccination (Biologic-exposed patients*)	−0.18	0.47	−0.29	0.26	−0.48	0.05

Spearman’s rank correlation coefficient was calculated to determine the relationship between two sets of non-parametric data. *Only includes women who received their last vaccination during pregnancy. **Bold**: indicates two-tailed p-value < 0.05.

In women who received their last vaccination during pregnancy, there was no correlation between gestational age at time of last vaccination and maternal nor cord blood antibody titers (Table 2). Both biologic-exposed women and all patients who were infected with SARS-CoV-2 during pregnancy had higher maternal and cord blood NT50, although this was only statistically significant for cord blood NT50 after correction for multiple comparisons (Fig 1C and not shown). There was no difference in neutralizing antibody titers between women treated with steroids during pregnancy and non-steroid-exposed women, both amongst women exposed to biologics (Fig 1D) and in all patients (not shown). As expected, there was a significant positive correlation between maternal and cord blood neutralizing antibody maternal titers (r = 0.96, p < 0.001, not shown).

### Placental antibody transfer

**Fig 2:**
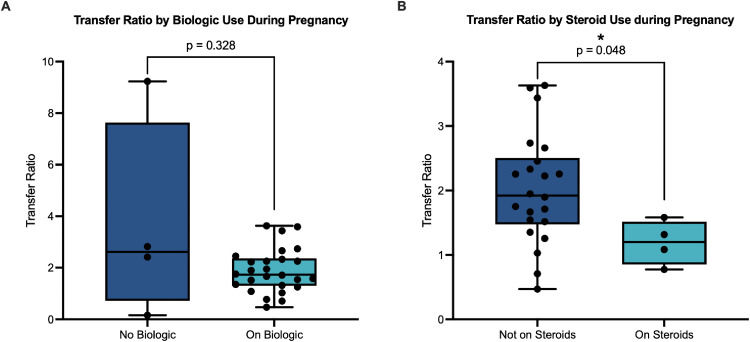
Exposure to biologics during pregnancy does not affect placental antibody transfer of anti-SARS-CoV-2 neutralizing antibodies, but concomitant corticosteroid exposure may decrease antibody transfer. (A) Anti-SARS-CoV-2 neutralizing antibody transfer ratio by biologic exposure during pregnancy. (B) Anti-SARS-CoV-2 neutralizing antibody transfer ratio by steroid use during pregnancy in biologic-exposed mothers. Mann-Whitney U tests were performed.

Placental transfer of neutralizing antibodies occurred in all biologic-treated women. As a measure of transfer efficiency, cord to maternal transfer ratios were calculated. There was no significant difference in transfer ratio based on biologic exposure during pregnancy (Fig 2A). However, a trend toward a higher transfer ratio was noted in the non-biologic group, although this is likely due to an outlier mother with a low neutralizing antibody titer yet a high cord blood titer, leading to a high transfer ratio. In biologic-treated women, the median transfer ratio was greater than 1.5 (1.73). In biologic-exposed women vaccinated during pregnancy, later gestational age at final vaccine dose was associated with a non-significant trend towards lower placental antibody transfer (Table 2). The negative correlation between transfer ratio and gestational age at time of final vaccination became statistically significant when including both biologic-exposed and non-exposed women (Table 2). Similarly, transfer ratio was positively correlated with vaccine latency among those vaccinated during pregnancy (Table 2). In biologic-exposed women, corticosteroid exposure during pregnancy was significantly associated with a lower placental transfer ratio, although this became non-significant after correction for multiple comparisons (Fig 2B and not shown). There was no significant correlation between maternal NT50 or cord blood NT50 and transfer ratio (not shown). There was no difference in transfer ratio between women who completed vaccination before pregnancy and those who completed it during pregnancy (not shown).

### Impact of immunomodulator therapy and vaccine type

Given the previously illustrated impact of immunomodulators on SARS-CoV-2 antibody titers, we performed a sensitivity analysis excluding immunomodulator-treated patients [3]. We found that the impact of timing of vaccination on maternal NT50 (i.e., before versus during pregnancy) and the effect of steroids on transfer ratio in biologic-exposed women became non-significant (S1A–B Fig). Similarly, these effects became non-significant when patients treated with mRNA-1273 vaccine and the BNT162b2 mRNA SARS-CoV-2 vaccine were analyzed separately (S1C–D Fig).

## Discussion

This study highlights important dynamics of neutralizing antibodies in pregnant women with IBD vaccinated against SARS-CoV-2. We found that biologic-exposed pregnant women with IBD were able to mount a neutralizing antibody response similar to their non-biologic-exposed counterparts. These results are in line with a prior study of anti-SARS-CoV-2 IgG levels in pregnant women with IBD, of which approximately half were on a biologic [9], but is in contrast to studies of non-pregnant patients with IBD on biologics, which have shown reduced levels of anti-SARS-CoV-2 IgG [3] and neutralizing antibodies [4] in infliximab-treated patients. Thus, pregnancy may be attenuating the negative impact of biologic exposure on antiviral immune response in these women.

Our study is the first to show robust placental transfer of anti-SARS-CoV-2 neutralizing antibodies in biologic-treated women, at rates similar to those of healthy women who were vaccinated during pregnancy reported in the literature (ranging from 0.77 to 2.6) [5,6,10]. These results also align with established transfer ratios for other vaccinations, such as measles, influenza, and pertussis, which all induce higher cord titers at delivery compared to maternal titers, with favorable (>1) transfer ratios of 1.2 to 3 [11].

Univariate analysis of our biologic-exposed cohort highlighted that mothers who completed SARS-CoV-2 vaccination before pregnancy had significantly higher neutralizing antibody titers at delivery than those who completed vaccination during pregnancy, and higher antibody titers with increasing vaccine latency, with the same trend in cord blood. Although most studies of SARS-CoV-2 IgG titers in women vaccinated during pregnancy have conversely demonstrated decreasing maternal and neonatal antibody titers further from vaccination, these studies did not examine women who were vaccinated before pregnancy. Therefore, our finding of increased neutralizing antibody titers in women vaccinated prior to pregnancy may reflect a combinatorial immunosuppressive effect of biologic exposure and pregnancy in women vaccinated during pregnancy, and/or simply a difference between neutralizing and non-neutralizing antibody dynamics, which has also been illustrated [11,12].

While we found no significant correlation between transfer ratio and vaccination latency in all-comers, and no difference in transfer ratio between those who completed vaccination before versus during pregnancy, we did observe a negative correlation between transfer ratio and gestational age at last vaccination in the subgroup of women who received their last vaccination during pregnancy, and similarly, a positive correlation between transfer ratio and vaccine latency in this subgroup. This is in line with previous findings that transfer ratio increases with increasing latency from vaccination to delivery in women vaccinated against COVID-19 during pregnancy, i.e., women vaccinated earlier in pregnancy demonstrate increased placental antibody transfer [6,11]. Therefore, there may be an advantage to COVID-19 vaccination earlier in pregnancy or before pregnancy to maximize the chances of robust placental antibody transfer.

Supporting a potential negative impact of immunosuppressive medications on placental antibody transfer, we found that corticosteroid exposure during pregnancy in biologic-exposed women significantly reduced transfer ratios. However, in our sensitivity analysis in which immunomodulator-treated patients were excluded, this finding became non-significant, suggesting that the combined use of different immunosuppressive agents (corticosteroids and immunomodulators) might have a cumulative impact on placental antibody transfer. Similarly, this finding also became non-significant in multiple comparisons analysis, reflecting the limitations of our small sample size. While previous studies have highlighted impacts of maternal factors such as chronic infection (including HIV, malaria), malnutrition, and metabolic disease on FcRn-mediated transplacental transfer [13], this is the first study to highlight a potential negative impact of corticosteroid use during pregnancy on placental antibody transfer in biologic and immunomodulator-exposed patients. Conversely, there was no impact of corticosteroid exposure during pregnancy on maternal nor cord blood neutralizing antibody titers, mirroring prior studies of maternal tetanus-diphtheria-and-acellular-pertussis (Tdap) vaccination in pregnant women on immune-modulating therapy for rheumatic disease, in which a subgroup analysis revealed anti-Tdap antigen IgG levels were similar between healthy and prednisone-treated subjects [14].

Consistent with prior studies of COVID-19 infection in non-vaccinated versus vaccinated pregnant women, we found that vaccinated mothers who were infected with COVID-19 during pregnancy exhibited higher neutralizing antibody titers, demonstrating a boosting effect of infection on the antiviral immune response in vaccinated pregnant women, although this finding was also non-significant after correction for multiple comparisons [10].

This study faces several limitations that impact the interpretability of its findings. The small sample size, particularly in the non-biologic cohort, restricts generalizability, making it difficult to isolate the effects of biologic versus immunomodulator exposure or vaccine versions. Furthermore, our study was found to be underpowered in a post hoc analysis, and thus true effects of biologic exposure on neutralizing antibody titers and placental antibody transfer may have gone undetected. Additionally, longitudinal measurements of neutralizing antibodies in mothers and infants after delivery could inform our understanding of the persistence and efficacy of these antibodies over time. Finally, incorporating newborn COVID-19 outcomes could provide additional context about the impact of maternal antibody transfer on neonatal health.

Overall, biologic treatment during pregnancy in women with IBD does not appear to impair the maternal or neonatal neutralizing antibody response to SARS-CoV-2, but combined steroid and immunomodulator exposure as well as vaccination timing may impact the placental transfer of neutralizing antibodies in this group. Future studies with larger cohorts and longitudinal follow-up are needed to validate these findings and assess if neutralizing antibody titers correlate with improved protection from COVID in biologic-exposed mothers and their neonates.

## Supporting information

S1 File Patient demographics, NT50 values, and transfer ratios. (XLSX)

S1 Fig Sensitivity analyses reveal the impacts of immunomodulator use and differential vaccine receipt. (A) Maternal NT50 by timing of SARS-CoV-2 vaccination completion in relation to pregnancy in biologic-exposed mothers, excluding immunomodulator-exposed mothers. (B) Anti-SARS-CoV-2 neutralizing antibody transfer ratio by steroid use during pregnancy in biologic-exposed patients, excluding immunomodulator-exposed patients. (C) Maternal NT50 by timing of SARS-CoV-2 vaccination completion in relation to pregnancy in biologic-exposed mothers, stratified by vaccine type. (D) Anti-SARS-CoV-2 neutralizing antibody transfer ratio by steroid use during pregnancy in biologic-exposed patients, stratified by vaccine type. Mann-Whitney U tests were performed.
(TIF)
